# Editorial: Retinitis pigmentosa, macular degeneration and related diseases

**DOI:** 10.3389/fncel.2026.1856506

**Published:** 2026-05-07

**Authors:** Ellen R. Weiss, Zongchao Han

**Affiliations:** 1 Department of Cell Biology and Physiology, University of North Carolina at Chapel Hill, Chapel Hill, NC, United States; 2 Department of Ophthalmology, University of North Carolina at Chapel Hill, Chapel Hill, NC, United States

**Keywords:** angiogenesis, macular degeneration, retinal degeneration, visual disease, retinitis pigmentosa

This special edition of Frontiers in Neuroscience describes novel investigations of visual defects and blindness caused by age-related macular degeneration (AMD) and retinitis pigmentosa (RP). Recent reports have indicated that AMD occurs in approximately 200 million people world-wide (Fleckenstein et al., 2021), making it the third leading cause of visual dysfunction after cataracts and glaucoma (Flaxman et al., 2017). There are 2 forms of (AMD): dry and wet. Dry AMD starts with the formation of aggregates known as drusen in multiple areas of the retina (Ambati and Fowler, 2012). This causes geographic atrophy in which areas of the retinal pigment epithelium (RPE) and the retinal photoreceptors begin to degrade (Ambati and Fowler, 2012), although dry AMD is less likely to cause severe visual impairment. In contrast, the retina undergoes choroidal neovascularization (CNV) in wet AMD, resulting in growth of new blood vessels that leak into the retinal space. Wet AMD is a more severe form and can cause total blindness. It is not clear whether these are different types of AMD or a progression of the disease from dry to wet over time (Ambati and Fowler, 2012).

A second form of retinal degeneration discussed in this topic is retinitis pigmentosa (RP). RP is an inherited genetic disease which, over time, results in the loss of rods causing night blindness. Ultimately, cones are also lost due to the lack of rod support, which results in complete blindness. Both dominant and recessive forms have been identified with different rates of degeneration. Mutations in over 90 genes have been implicated in RP (Nguyen et al., 2023). This high variance in the causes of RP suggests that gene therapy will not be effective for the majority of patients although one form of RP, caused by mutations in RPE65, has been treated with the drug Luxturna, which is commercially available (Nguyen et al., 2023; Maguire et al., 2019).

The aim of this Research Topic is to define the prevalence of AMD and RP in recent global surveys in coordination with associated risk factors, and identify potential avenues for treatment.

## Reviews

A 2021 update to a study of the Global Burden of Disease, which can be found in the Global Health Data Exchange Database (https://vizhub.healthdata.org/gbd-results/), provides an analysis of factors concurrent with the prevalence of non-communicable diseases, including multiple causes of blindness. These vision-related diseases included cataract formation, glaucoma, diabetic retinopathy and AMD. The collected data span the years 1990–2021 and covers 204 countries and 811 subnational locations (Ferrari et al., 2024).

Using this database, Ye et al. evaluated the data for factors that appear to coincide with vision loss, and ultimately, blindness, at a global level. These factors included population characteristics such as nation, age, gender, socioeconomic status and the quality of healthcare. The purpose of this analysis was to predict the prevalence of disease in 2045 and identify factors related to healthcare that can be altered to mitigate the predicted increases. The severity of blindness and vision loss (BVL) was found to have increased significantly in the past 20 years in both males and females, although females were found to have a greater increase, possibly due to less access to healthcare. Countries with the lowest levels of health care contributed disproportionately to this increase, particularly for diseases such as AMD. Recommendations aimed to reduce the levels of BVL in affected countries are discussed.

A second manuscript using these data by Kong et al. selectively focuses on factors contributing to the global prevalence of AMD. Similar to the previous report, higher levels and greater severity of disease were noted among females and in areas with the lowest socio-economic index (SDI). It was also observed to develop at a younger age. AMD increased globally over 20 years by approximately 121%. Individuals living in regions with lower socio-economic status were found to have the highest prevalence of AMD. Interestingly, age-standardized prevalence decreased over the period studied while the number of years living with disability increased. This is possibly due to earlier detection and an increase in the use of anti-VEGF, which slows the advance of disease, in wealthier countries. A reduction in disease-related activities such as smoking, a known risk factor for AMD, was also noted (Seddon et al., 2006).

## Research papers

An ongoing issue in quantifying vision loss in patients is the subjectivity and non-quantitative nature of the tests. For example, visual acuity and standard automated perimetry (SAP) require patient input, which depends on cognitive ability, patience and cooperation and tends to be highly variable (Heijl et al., 2022; Mendieta et al., 2021; Spry et al., 2001). Trinquet et al. proposed a novel method for measuring AMD-dependent vision loss using multifocal Pupillary Response Field (mPRF) analysis. The test examines 9 areas of the retina simultaneously, resulting in more comprehensive testing in a shorter time period and requires no input from the patient, eliminating the subjectivity of the test. Each test requires only 1 min per replicate compared to standard methods which require 5–10 min of dark adaptation followed by testing periods interspersed with recovery so that multiple regions of the retina can be tested (Kelbsch et al., 2020, 2019). The results suggest that pupillometry is an excellent substitute for present methods of analysis. No dark adaptation period is required and a geographically broad analysis can be performed in a short time period. Future studies will address whether this method is suitable for long-term studies in individual patients and whether responses to therapeutic interventions can be measured.

In a report by Yi et al., aspirin was found to enhance nAMD in model systems. Mice were given low dose aspirin or vehicle for 8 weeks, after which CNV was induced by laser treatment. A decrease in thrombospondin-1 (TSP-1), which is involved in wound-healing, was detected. These mice were observed to have more severe CNV than controls not treated with aspirin. Patients with nAMD were found to have lower serum levels of TSP-1 than individuals with no AMD although aspirin use did not appear to affect these levels. The link between aspirin and AMD in patients is highly controversial due to other risk factors such as cardiovascular disease, diabetes and the presence of other medications. The data provided in this manuscript indicates that low TSP-1 levels may be a risk factor for nAMD. Future studies will evaluate the effect of aspirin and the levels of TSP-1 in AMD patients in more comprehensive randomized controlled studies.

As described above, RP is a genetic disease primarily affecting rods and leading to total blindness when cones degenerate as a secondary consequence of rod degeneration. Yang et al. evaluated the effect of injecting human retinal progenitor cells (RPCs) intravitreally in patients to determine safety and tolerability. Two groups of patients with different levels of blindness were evaluated in this Phase I/IIa safety trial. Donated tissue underwent transcriptome and protein expression analyses to identify RPC progenitor cells with low levels of specific antigens that might cause an immune reaction in the recipients. Patients were examined at 4 weeks for adverse events, then re-evaluated at 6 months and 1 year for changes in vision using standard techniques. The results indicate an improvement in visual acuity in the injected eye, particularly in patients who received the highest dose of RPCs. No serious adverse events or graft rejection were detected, indicating that this is a promising therapeutic approach for patients with blindness due to rod cell degeneration. The patients will continue to be followed. Additional studies to further identify appropriate doses for effective restoration of visual function will be performed.

In summary, the work presented in this Research Topic presents updated information on conditions that influence the world-wide prevalence of AMD and RP and the severity of these diseases under different socio-economic regions. In addition, improved methods for evaluating blindness, potential risk factors and possible treatments are described.
